# Serum Nickel and Titanium Levels after Transcatheter Closure of Atrial Septal Defects with Amplatzer Septal Occluder

**DOI:** 10.1155/2019/7891746

**Published:** 2019-01-02

**Authors:** Ozlem Elkiran, Cemsit Karakurt, Gulendam Kocak, Cagatay Taskapan

**Affiliations:** ^1^Department of Pediatric Cardiology, Faculty of Medicine, Inonu University, Malatya, Turkey; ^2^Department of Pediatric Cardiology, Medical Park Hospital, Istanbul, Turkey; ^3^Department of Biochemistry, Inonu University Faculty of Medicine, Malatya, Turkey

## Abstract

**Introduction:**

There is a concern about release of nickel and titanium after implantation of nitinol-containing devices.

**Objective:**

To evaluate serum nickel and titanium release after implantation of Amplatzer occluder.

**Materials and methods:**

In 38 pediatric patients with no history of nickel sensitivity, blood samples were drawn 24 hours before and 24 hours, 1, 3, 6, and 12 months after implantation. Nickel and titanium concentrations were measured by atomic absorption spectrophotometry.

**Results:**

The median serum nickel level which was 0.44 ng/mL before the implantation increased to 1.01 ng/mL 24 hours after implantation and 1.72 ng/mL one month after implantation. The maximum level was detected 3 months after implantation, with a median level of 1.96 ng/mL. During follow-up, the nickel levels decreased to those measured before implantation. Serum nickel levels at the 24th hour, 1st month, and 3rd month following implantation were found to have increased significantly. No patients showed a detectable serum titanium level.

**Discussion:**

This is the first study that evaluated both serum nickel and titanium release after implantation of the Amplatzer occluder. Our study shows that nickel is released from the device in the first few months after implantation. Therefore, in patients with nickel allergy, other devices may be considered.

## 1. Introduction

Secundum-type atrial septal defects (ASD) account for about 6%–10% of congenital heart defects in children [1, 2]. Untreated hemodynamically important atrial septal defects can cause right ventricular overload with right heart failure, atrial arrhythmias, systemic embolism, pulmonary hypertension, and death. Although results of surgical closure are good, they are associated with morbidity, discomfort, and thoracotomy scar. Currently, transcatheter closure of ASD has become a method that is preferred for open heart surgery. In contrast to surgical repair, the transcatheter approach avoids cardiopulmonary bypass, results in shorter hospitalization, reduces need for blood products, and lessens patient discomfort [3–6].

The Amplatzer septal occluder (AGA Medical Corp, Golden Valley, MN; USA) is a nickel-titanium alloy (nitinol) device which is the commonly implanted device for the transcatheter closure of ASD and produces good early-, middle-, and long-term results in children and adults [6–10]. Nitinol is an alloy composed of nickel and titanium. It is used commonly in medical products due to its good radio-opacity, superelasticity, corrosion resistance, and shape memory quality [11, 12]. However, concerns have been raised about the potential release of nickel and titanium after implantation of nitinol devices. Systemic side effects associated with nickel allergy, pericarditis, and increased migraine headaches have been reported in patients with transcatheter closure of interatrial shunts [13–15]. Moreover, research has also demonstrated that nickel is cytotoxic and carcinogenic to humans [16–19]. Titanium is an inert metal that has been used increasingly commonly in orthopedic devices and oral implants and is believed to display a high degree of biocompatibility. However, it can cause allergic and toxic effects in blood, fibrotic tissues, and osteogenic cells [20]. These reasons make it all the more important to know the levels of nickel and titanium releasing from the commonly used Amplatzer device, particularly in children and young adults. The present study aimed at examining the serum nickel and titanium release after transcatheter implantation of Amplatzer device that is commonly used.

## 2. Materials and Methods

This study was conducted in the Department of Pediatric Cardiology of Inonu University Medical School. The parents of all the children were informed about the study, and their written consents were taken. In accordance with the Helsinki Declaration, the approval of the Ethics Committee of Inonu University Medical School was acquired before the study. Project identification code: 2010–153.

### 2.1. Patient Selection

The study included 38 pediatric patients (14 males and 24 females) with no history of nickel sensitivity, those who have undergone percutaneous closure of ASD with the Amplatzer septal occluder. The demographic and clinical characteristics of the patients were recorded. All patients were evaluated at our institution with transthoracic two-dimensional and color Doppler echocardiography using a Vivid Pro 7 (GE Vingmed Ultrasound, Horten, Norway) echocardiography device. Indications for closure were a significant left-to-right shunt, pulmonary and systemic blood flow (Qp/Qs) ratio >1.5, volume overload of the right ventricle, and development of symptoms. The selection of patients suitable for a transcatheter closure was based on the morphology and location of the defect and presence of rims around the defect. Patients who had sinus venosus and ostium primum type atrial septal defect, other congenital heart defects that require surgical repair, severe pulmonary hypertension, as well as patients with more than two rim deficiencies and septal rims <5 mm from the right pulmonary vein, coronary sinus, superior vena cava, inferior vena cava, coronary sinus, and atrioventricular valves were excluded from the study.

### 2.2. Procedure and Device

All the procedures were performed under general anesthesia. Venous access was gained via the femoral vein. Right and left heart catheterization was performed to measure the pulmonary artery pressure and to calculate pulmonary vascular resistance with left-right shunt volume. Pulmonary to systemic flow (Qp/Qs) ratio was calculated by oximetry according to Fick's principles. Multiplane transesophageal echocardiography (TEE) was performed before the implantation for detailed assessment of the size, location, and relation of the ASD to surrounding tissues. Using fluoroscopic and TEE guidance, the Amplatzer septal occluder was implanted using previously reported techniques [21]. The Amplatzer septal occluder is composed of nitinol (Ni amount 54.5–57.0 wt.%, balance Ti). Nitinol wire is medical grade per requirements set forth by ASTM F2063 (Standard Specification for Wrought Nickel-Titanium Shape Memory Alloys for Medical Devices and Surgical Implants). The device is sterilized by Ethylene Oxide (EtO) method. To increase the occlusive capacity and to ensure a rapid neoendothelial development, each disc was filled with polyethylene terephthalate (Dacron). The nitilol wire goes through a proprietary chemical etching process at the vendor. No other surface treatment and coating is carried out at the manufacturing site [7, 22].

### 2.3. Laboratory Analysis

Blood samples for serum nickel and titanium analysis were collected before implantation and on day 1 and in months 1, 3, 6, and 12 after the implantation. The blood was centrifuged immediately, and the serum was frozen at −20°C until analysis was performed. Serum nickel and titanium analyses were conducted by atomic absorption spectrophotometry with the Perkin-Elmer atomic absorption spectrophotometer Analyst 800 (Bodenseewerk Perkin-Elmer & CoGmbH, Uberlingen, Germany) and THGA graphite tubes (Perkin-Elmer & CoGmbH). Serum levels of nickel <2 ng/mL were considered to be normal.

### 2.4. Ethical Standards

The parents of all the participating children were informed about the study, and their informed consent was received. In accordance with the Helsinki Declaration of 1975, as revised in 2008, the Ethics Committee of the institute approved the study before it began.

### 2.5. Biostatistical Analysis

The data were expressed as either median (25%–75%) values depending upon overall variable distribution. Normality was assessed using Shapiro-Wilk test. The nonnormally distributed data for repeated observations were compared by Friedman test. When significant differences were determined, multiple comparisons were carried out using Bonferroni-corrected Wilcoxon test. *p* value < 0.05 was considered statistically significant. IBM SPSS statistics version 25.0 for Windows was used for statistical analyses.

## 3. Results

At the time of implantation, the median patient age was 6 (range 3–16) years and weight was 17 (range 10–61) kg. The median size of the implanted occluder was 13 (range 8–24) mm. The device was implanted successfully in all patients. There were no deaths, cardiac perforations, device embolizations, significant arrhythmias, infective endocarditis, or other morbidity associated with the procedure during the entire follow-up period.

**Figure 1:**
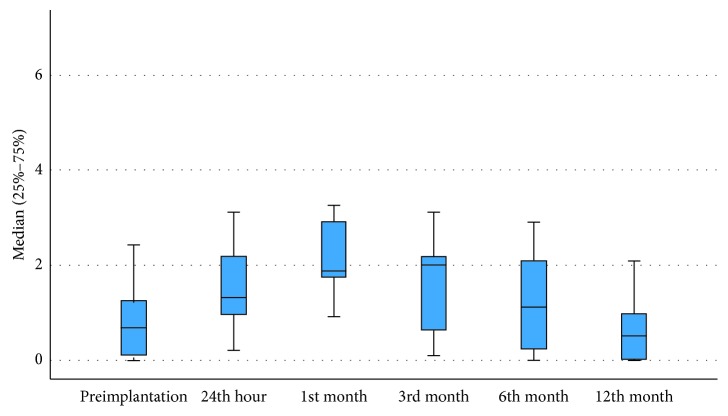
Median serum nickel level at six different time points during the study period.

The median serum nickel level which was 0.44 ng/mL before the implantation, increased to 1.01 ng/mL 24 hours after implantation and 1.72 ng/mL one month after implantation. The maximum level was detected 3 months after implantation, with a median level of 1.96 ng/mL. Later, the median serum nickel level gradually dropped after the implantation to 1.08 ng/mL in month 6 and 0.52 ng/mL in month 12 (Figure 1). When compared to baseline values, serum nickel levels at the 24th hour, 1st month, and 3rd month following implantation were found to have increased significantly (*p* < 0.0001) (Table 1). The range of nickel levels was between 0 and 9 ng/mL at all times. There was a trend to higher serum levels of nickel in larger devices and it was statistically significant (Table 2). None of the patients showed a detectable serum titanium level. None of the patients were found to develop any toxic or allergic reactions associated with elevated nickel during the follow-up period.

## 4. Discussion

To our knowledge, this is the first study that evaluated both serum nickel and titanium release in children after transcatheter closure of ASD with Amplatzer septal occluder. Corrosion behavior of nitinol in the body is of critical importance due to the possible toxic effects of nickel and titanium. The corrosion of the metal in the implanted material is a complex process which takes place due to the corrosive environment in the body. None of the metallic materials in living tissues are entirely resistant to corrosion. Over time, a protective oxide layer is formed on surface of the metals and alloys used as implants. This layer inhibits corrosion and limits the release of metal ions. [19, 23–25].

Studies exploring nickel release in devices that contain nitinol have produced inconsistent results. Several studies have shown that there is in vivo and in vitro nickel release and reported nickel allergy in patients in whom interatrial shunts were closed with nitinol-containing devices [26–28]. Ries et al. followed a total of 67 patients in whom the Amplatzer septal occluder was used for transcatheter closure of ASD and patent foramen ovale for a period of one year and found a statistically significant elevation, relative to the baseline values, in mean nickel levels 24 hours and 1 month after implantation [7]. In the concerned study, the highest nickel level was established in the first month following implantation. However, it was reported that some patients already had high serum nickel levels, due to some unknown cause, before the implantation. None of the patients experienced any side effects or showed any allergic signs associated with nickel allergy over the follow-up period. Burian et al. measured serum and urine nickel levels in a total of 24 patients who had undergone transcatheter ASD closure and reported that serum nickel levels showed a 5-fold increase in comparison to baseline values in week 6 after implantation and that serum and urine nickel were restored to their normal levels in 4 to 6 months after implantation [29]. None of the patients had any complications throughout the 12-month follow-up. It was concluded on the basis of these results that the nickel release from the Amplatzer device in the beginning did not pose any specific cardiovascular risk. The median serum nickel level was found statistically elevated relative to the baseline level 24 hours, 1 month, and then 3 months after the implantation in our study. The highest median nickel level was detected in months 3 after implantation, after which nickel gradually decreased to its baseline level. In line with the above studies, there was no side effect associated with nickel intoxication in any of our patients.

In contrast to the cited studies, it has been reported in some publications that there is no nickel release following the implantation of nitinol-containing devices. Kong et al. demonstrated in their study that nitinol used in the Amplatzer septal occluder devices was resistant to corrosion, when it was exposed to a physiologic saline solution [22]. In the concerned study, serum nickel levels in 19 patients to whom the Amplatzer device was implanted were measured, and no increase in nickel levels was found after the procedure. Based on these results, it was concluded that nickel-titanium compounds were inert and resistant to corrosion. The number of patients in whom serum nickel levels were measured in this study was only 19, which may be too low to evaluate nickel release. Assad M et al. evaluated that the genotoxicity level of nickel‐titanium was compared to that of its pure constituents, pure nickel, and pure titanium powders and also to 316L stainless steel. In this study, a dynamic in vitro semiphysiological extraction was performed all metals. Peripheral blood lymphocytes were then cultured in the presence of all material extracts, and their comparative genotoxicity levels were assessed using electron microscopy. Additionally, the graphite furnace atomic absorption spectrophotometry was also performed on all extraction media. Based on these results, it was reported that both pure titanium and nickel‐titanium specimens obtained a relative in vitro biocompatibility and suggested optimistic results for the eventual use of nickel‐titanium alloys as surgical implant materials [30].

There were individual patients who were found to have elevated serum nickel levels both before and after implantation in our study. In the study period, only a few patients were found to have serum nickel at the maximum level at different times. Besides, there were also patients whose serum nickel did not increase throughout the follow-up. Although what causes this discrepancy is not known for sure, it is believed that there may be multiple contributing factors. For instance, individual differences in endothelialization process or personal factors in the formation of the oxide layer may alter nickel release. As with other metals used in implanted devices, the corrosion characteristics of nitinol-containing devices, the localization of the implant and environmental conditions may influence the biological reactions of these devices. Some authors claimed that localized mechanical stress or friction caused by the movement of the heart could damage the surface layer on the device and that the duration of nickel release might be extended until endothelialization around the device was complete [7]. Additionally, the corrosion resistance of nitilol-containing devices seems to depend on the surface properties of the alloy. For this reason, many surface treatments have been considered. Villermaux et al. investigated using of the laser surface melting to improve the corrosion resistance of nitilol alloy and reported that the corrosion behavior of the nitilol sample could be improved by laser melting surface [31]. In another study performed by Cissé et al., the effect of surface modification on corrosion behavior of nitilol was investigated and found that the lowest corrosion rate was obtained on chemical passivation and the highest one was obtained on mechanical polishing [32]. Also, since the possible nickel release from nitilol-containing septal occluders is reported in the literature, some of the produced septal occluders are coated with golden-yellow titanium or ceramic. The companies propose that these properties decrease nickel release into the blood and endocardium and thus reduce nickel toxicity [33, 34].

Although there is no correlation between the serum nickel level and allergic reactions, nickel released from the implant may trigger allergic reactions, and this possibility persists for three months after implantation. The duration of nickel release is an important factor with regard to potential allergic reactions. It may be that nickel release is inhibited in the later periods after the formation of the stable oxide layer on the device surface. In our study, no allergic or toxic reactions were observed.

Although titanium has been considered an inert and biocompatible metal for a long time, its biocompatibility has recently become a matter of concern. Several studies have shown that titanium has potential hematological and metabolic toxic effects [20, 23]. Despite the fact that titanium-containing alloys are used in some artificial joints, prosthetic devices, and implants, the nature of titanium as well as the implications of its systemic effects are not known for certain. Studies examining systemic titanium release have presented disparate results. It has been claimed that titanium released from implanted devices could be spread to the body through the circulatory or lymphatic system, causing harmful effects on the blood, fibrous tissues, or osteogenic cells [20, 23]. In a study where the patients who had undergone primary total artificial hip implantation containing titanium were followed for 120 months, a significant increase was established in serum titanium levels. It was argued in the concerned study that the toxicological risks associated with metal ions represent an area of much-needed investigation [35]. In the same vein, Jacobs et al. reported a three-fold increase in the concentration of titanium in the serum of patients in whom joint arthroplasty is performed using titanium-containing implants [36]. In patients in whom hip and knee arthroplasty was performed, titanium alloy particles were identified in tissues lymph nodes and some remote organs [37].

In contrast, Bianco et al. who evaluated the titanium levels in the serum and urine of rabbits which had titanium implants found that the titanium levels in this group were not different than those in the control group [38]. Similarly, no detectable titanium was found in any patient throughout the follow-up period in our study. This result indicates that there is no detectable titanium release from the Amplatzer device. Thus, it can be argued that titanium is more resistant than nickel to corrosion and the possible localized stress and friction caused by the motion of the heart.

## 5. Conclusion

It was showed in our study that although there was nickel release from the Amplatzer device in the first few months after implantation, none of the patients had any toxic or clinical adverse effects during the follow-up. However, there was no detectable titanium release in our patients.

Although the implantation of nitinol-containing devices such as the Amplatzer septal occluder seems to be a very reliable method from a technical point of view, it is of utmost importance to know and monitor the possible metal release from these devices and the associated adverse effects.

The results of our study suggest that both metals are biocompatible. However, obtaining a detailed history of nickel allergy and even conducting a nickel allergy test in patients to whom a nitinol-containing device will be implanted is important to prevent possible side effects. In patient with nickel allergy, coated devices may be considered if available.

## Figures and Tables

**Table 1:** Statistical evaluation of serum nickel levels.

Variable	Median	Percentile 25	Percentile 75	^*∗*^ *p* value
Preimplantation	0.44	0.00	0.82	<0.0001
24th hour	1.01^‡,§^	0.30	2.04	
1st month	1.72^‡,§^	0.96	1.98	
3rd month	1.96^‡,§^	0.54	2.18	
6th month	1.08^¶^	0.05	2.10	
12th month	0.52	0.00	1.04	

The nonnormally distributed data for repeated observations were compared by Friedman test. When significant differences were determined, multiple comparisons were carried out using Bonferroni-corrected Wilcoxon test. ^‡^Significantly different from preimplantation; ^§^Significantly different from 12th month; ^¶^Significantly different from 1st month (*p* < 0.001; Bonferroni-corrected Wilcoxon test); ^*∗*^*p* value of the Friedman test.

**Table 2:** The correlation coefficient (*r*) and probability (*P*) values between device diameter and serum nickel levels.

Pre	Preimplantation	24th hour	1st month	3rd month	6th month	12th month
Device diameter						
*r*	0.438	0.642	0.728	0.832	0.658	0.669
*P*	0.006	0.000	0.000	0.000	0.000	0.002

## Data Availability

All data generated or analysed during this study are included in this article.
